# Pre‐Operative Intraoral Ultrasound Features of Benign and Malignant Oral Lesions: An Exploratory Pilot Study

**DOI:** 10.1111/odi.70395

**Published:** 2026-06-18

**Authors:** Ragda Abdalla‐Aslan, Diana Gaitini, Adi Rachmiel, Goni Merhav, Sharon Akrish, Marcia Javitt, Dekel Shilo, Omri Emodi, Yotam Shkedy, Nira Beck‐Razi

**Affiliations:** ^1^ Rappaport Faculty of Medicine Technion Israel Institute of Technology Haifa Israel; ^2^ The Department of Oral and Maxillofacial Surgery Rambam Health Care Campus Haifa Israel; ^3^ The Department of Medical Imaging Rambam Health Care Campus Haifa Israel; ^4^ The Department of Pathology Rambam Health Care Campus Haifa Israel; ^5^ Department of Radiology University of Miami Miller School of Medicine Miami Florida USA; ^6^ Department of Radiology Mount Sinai Medical Center Miami Beach Florida USA; ^7^ Department of Otolaryngology‐Head and Neck Surgery Rambam Health Care Campus Haifa Israel

**Keywords:** cancer prediction, diagnostic imaging, diagnostic ultrasound, mouth neoplasms, oral squamous cell carcinoma

## Abstract

**Objective:**

To evaluate intraoral‐ultrasound (ioUS) features of benign and malignant oral lesions.

**Methods:**

In this prospective cross‐sectional study, patients with oral soft tissue lesions underwent ioUS followed by biopsy within 2 weeks. Sonographic features: size, echogenicity, cystic areas, calcifications, margins, and vascularity were compared with histopathology. Associations between sonographic features and malignancy were explored using univariate and multivariable analyses. ROC analysis was used descriptively to explore discriminatory ability.

**Results:**

Fifty‐one patients with 52 lesions (24 females, 27 males; mean age 60.1 ± 16.7 years, range 18–90) were recruited. Following exclusions, the study comprised 23 histologically confirmed malignant cases (squamous cell carcinoma, SCC) and 23 benign cases. Multivariate analysis identified maximal diameter (> 12 mm; sensitivity 0.76 [95% CI: 0.53–0.90], specificity 0.77 [95% CI: 0.57–0.90]), ill‐defined margins (*p* < 0.001), and increased vascularity (*p* = 0.002) as being associated with SCC in this cohort. Combinations of these features showed high apparent discrimination (AUC 0.92 [95% CI: 0.84–1.00]), but likely reflect optimistic bias given the small sample size and lack of validation.

**Conclusions:**

This pilot study describes ioUS features in benign and malignant oral lesions and identifies sonographic features potentially associated with malignancy. These findings are exploratory and hypothesis‐generating and require validation in larger, more diverse cohorts before any clinical application.

## 1 Introduction

Oral squamous cell carcinoma (SCC) accounts for 90%–95% of oral cavity cancer, with significant mortality and long‐term morbidity (Jerjes et al. 2010). Prognosis depends on stage at presentation and the site of involvement, with an overall 5‐year survival rate of approximately 50% (Bjerkli et al. 2020; Listl et al. 2013; Zini et al. 2010). Because of its ambiguous clinical presentation, diagnosis of early stage oral SCC is challenging and is frequently misdiagnosed, mistreated, and linked to diagnostic delays (Walsh et al. 2021).

A definitive diagnosis between benign and malignant lesions is based on biopsy and pathologic finding; however, these procedures are invasive, time‐consuming, and may result in post‐operative complications (Avon and Klieb 2012; Emperumal et al. 2024; Logan and Goss 2010; Okuyama et al. 2018; Valdez et al. 2022). In addition, there are implications for patient compliance, especially when several or repeated biopsies are required for follow‐up (Emperumal et al. 2024). Biopsy remains the gold standard for diagnosing persistent oral lesions. However, non‐invasive adjunctive tools such as intraoral ultrasound (ioUS) may provide valuable noninvasive pre‐biopsy information to guide clinical decision‐making and prioritize cases.

Currently, clinicians rely mainly on clinical features when assessing oral lesions: size, induration, ulceration, bleeding, and pain may suggest malignant lesions. A systematic review found that clinical examinations can diagnose early oral SCC in otherwise healthy individuals with an accuracy of only 1% to 51% (Walsh et al. 2021). There is a clear and present need for the development of innovative noninvasive methods for the identification of oral cancer, such as vital staining, tissue autofluorescence, and optical coherence tomography (Romano et al. 2021). However, none of these methods are widely accepted or implemented in clinical practice (Romano et al. 2021). Magnetic resonance imaging (MRI) is safe and widely used to assess oral lesions, but is limited by availability, cost, and artifacts due to dental metals (Bohner et al. 2022; Gao et al. 2022; Shafiei et al. 2003; Tunlayadechanont et al. 2024).

Ultrasound (US) is a widely utilized noninvasive imaging modality with many advantages. US is useful for discriminating benign and malignant lesions in soft tissues (Takeshi Morii et al. 2018; Shu et al. 2022), as well as in other tissues such as the breast (Du et al. 2012) and thyroid (Pei et al. 2019). In the oral cavity, the use of ioUS has been reported for assessing benign lesions and hemangiomas (Derindağ et al. 2021; Konishi et al. 2023), as well as for dental indications including periodontal disease and dental implantology (Renaud et al. 2023; Rodriguez Betancourt et al. 2023). Chan and colleagues have made important contributions to this field by establishing key imaging principles and intraoral scanning techniques, and by demonstrating the potential of ioUS to evaluate facial crestal bone, periodontal soft tissues, and peri‐implant structures, with quantitative assessments of tissue morphology that are comparable to conventional imaging modalities such as cone‐beam computed tomography (CBCT) (Bhaskar et al. 2018; Chan et al. 2017). In addition, ioUS has been shown to be beneficial for determining the depth of invasion in and tumor thickness in tongue SCC (Esperouz et al. 2024; Tarabichi et al. 2019). Studies by Izzetti and colleagues have demonstrated that ultra–high‐frequency ultrasound (UHFUS) can reliably differentiate normal from pathological oral mucosa and assess mucosal growths, autoimmune diseases, potentially malignant lesions, and oral SCC (Izzetti, Vitali, Aringhieri, et al. 2020a; Izzetti, Vitali, Aringhieri, Oranges, et al. 2021). Key sonographic features evaluated included mucosal thickness, echogenicity, and vascularity. Vascular patterns further aided lesion characterization, with mild to moderate vascularity suggesting benign or inflammatory pathology and marked vascularity more frequently observed in premalignant and malignant disease (Izzetti, Vitali, Aringhieri, et al. 2020b; Izzetti, Vitali, Aringhieri, Oranges, et al. 2020). UHFUS operates at higher frequencies (30–100 MHz) compared with conventional ioUS (10–15 MHz), providing exceptional spatial resolution (up to 30 μm) but is limited by a shallow penetration depth of approximately 1 cm from the surface (Fogante et al. 2022). Its widespread clinical adoption is limited due to restricted availability, with use primarily concentrated in specialized tertiary centers. In contrast, conventional ioUS offers greater tissue penetration, making it better suited for assessing submucosal and deeper lesions, and its wider availability enhances clinical applicability in routine practice.

A study by Joshi et al. included 10 patients with cancerous growths, including bony tumors and a neck mass, and used a low‐frequency endocavity probe (Joshi et al. 2014). The authors reported that sonographic examination suggested malignancy; however, specific imaging features leading to this assessment were not clearly described. Among the seven oral SCC lesions, the tumors were generally hypoechoic and infiltrative. A recent scoping review evaluated 34 studies on the diagnostic potential of US for oral lesions, using MRI, CT/Cone‐beam CT, or histopathology as reference standards (Pacheco‐Pereira et al. 2025). The majority of studies focused on malignancies, primarily reporting the established correlation between tumor thickness or depth of invasion to their pathological or imaging counterparts, while only one study assessed soft tissue lesions (using UHFUS rather than conventional ioUS) and another investigated intraosseous lesions. Overall, US demonstrates promise as a reliable, non‐invasive method for characterizing both benign and malignant oral lesions (Pacheco‐Pereira et al. 2025). Collectively, these studies highlight the growing versatility of ioUS assessment of oral soft tissue lesions.

This prospective study aimed to evaluate preoperative ioUS features in both benign and malignant oral lesions, and to investigate whether specific combinations of sonographic findings might correlate with malignancy. Our working hypothesis was that certain ioUS characteristics are potentially correlated with malignancy, serving as a basis for further hypothesis generation.

## 2 Methods

### 2.1 Ethical Considerations

The study was approved by the institutional review board (RMB‐19‐0596). Patients signed informed consent to participate and could withdraw at any stage. Each participant received a serial number, and all personal identifiers were removed to ensure anonymity. Participants received treatment as considered appropriate for their diagnosis, regardless of the study.

### 2.2 Study Design and Population

This was a prospective study of consecutive patients who were enrolled from June 2021 until December 2023. Inclusion criteria were patients older than 18 years of age from both genders with oral mucosal lesions who are scheduled for biopsy and histopathological examination. The patients underwent ioUS after signing informed consent, within a 2 week interval before the planned biopsy. Exclusion criteria were patients with limited mouth opening, images with poor quality and/or artifacts, and non‐diagnostic or non‐specific pathological results.

The intended sample size was based on the number of eligible patients who agreed to participate within the study period. As an exploratory pilot study, no formal power calculation was performed. In the absence of established ioUS criteria for oral lesions, the sample size (*n* = 46; 23 malignant, 23 benign) was determined pragmatically based on case availability. This approach aligns with guidance suggesting that pilot studies should prioritize study objectives and descriptive precision over formal power, with 30–50 subjects typically considered appropriate (Hertzog 2008; Kunselman 2024). According to the Viechtbauer et al. (2015) framework, 23 cases per group provides 95% confidence in identifying features with at least a 12.2% occurrence probability. Diagnostic accuracy is reported with confidence intervals to reflect the precision of these preliminary findings.

### 2.3 Intraoral Ultrasound

High‐resolution ioUS images were obtained using a 15 MHz L15‐7io linear ultrasound transducer “hockey stick probe” on a Philips Epiq 5 and 7 machine (Philips Medical) with a 23‐mm rectangular field of view and a single near‐field focal zone. All examinations were performed and interpreted by one radiologist (NBR), with more than 15 years of experience in general US. The probe was placed in a sterile cover and saliva combined with commercial oral gel used as a coupling agent. Saliva alone is usually not adequate as coupling agent and the use of commercial oral gels is recommended (such as Biotene oral balance gel) to prevent soft tissue compression, to ensure consistent image quality and compliance with infection control requirements, as the acoustic properties of coupling media can influence signal transmission and image quality, and adequate acoustic coupling is essential for reliable ioUS imaging (Kripfgans and Chan 2023). The probe was gently placed on the lesion so as not to distort the mass and slided along the mucosal surface, as shown in detail by an earlier investigation (Abdalla‐Aslan et al. 2022). The following US features of each mass were recorded (Figure 1a–i):

1. Maximal lesion's dimensions in millimeters in three dimensions‐ length, width and depth.
2. Margins: well defined, poorly defined
3. Echotexture: hyperechoic, hypoechoic, mixed echogenicity.
4. Content: solid; presence of cystic areas
5. Presence or absence of calcification
6. Vascularity (using color Doppler US, Figure 1j–m):
  - Yes; No
  - According to Giovagnorio's criteria (Giovagnorio et al. 1999): Type I: avascular (no detectable blood flow within or around the lesion on Doppler imaging); Type II: hypovascular (few scattered or faint vessels); Type III: hypervascular with multiple peripheral vessels (multiple vessels predominantly at the lesion periphery); Type IV: hypervascular with internal vessels (marked intralesional vascularity with vessels distributed within the lesion).

**FIGURE 1:**
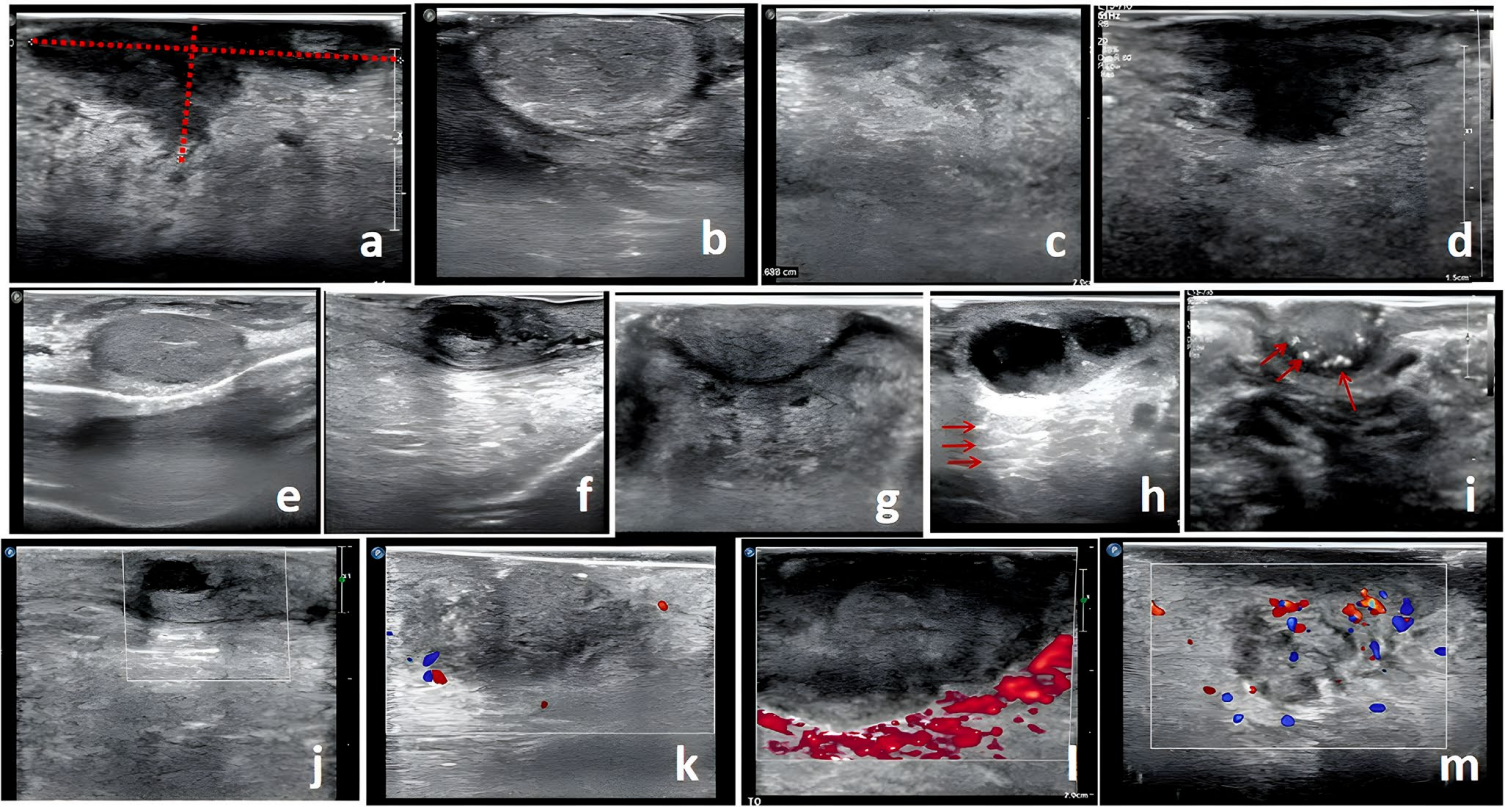
Sonographic variables assessed in the study: Dimensions of the lesion (a); Borders: Well‐defined (b) or ill‐defined (c); Ecotexture: Hypoechoic (d), hyperechoic (e), or mixed (f); Content: Solid lesion (g) or lesion with cystic areas (h, note posterior acoustic enhancement—arrows) and presence of calcifications (i, arrows). Vascularity according to Giovagnorio's criteria: Type I: Avascular (j); Type II: Hypovascular (k); Type III: Hypervascular with multiple peripheral vessels (l); Type IV: Hypervascular with internal vessels (m).

ioUS acquisition and interpretation were performed by a single board‐certified radiologist (MD). While the radiologist had prior experience in conventional head and neck US, they had no previous exposure to ioUS or any expertise in characterization of oral mucosal lesions. To ensure diagnostic independence, the radiologist remained blinded to clinical impressions from oral medicine specialists. Furthermore, histopathological results were unavailable to both the radiologist and treating clinicians at the time of examination, as all biopsies were performed subsequent to the ioUS assessment.

Features of ioUS were compared to histopathology serving as the reference standard. All tissue specimens were immediately fixed in 10% neutral buffered formalin, routinely processed, and embedded in paraffin. Sections (3–5 μm) were stained with hematoxylin and eosin (H&E) for microscopic evaluation, with ancillary stains applied when required for diagnostic clarification, including immunohistochemical markers for SCC (e.g., cytokeratins AE1/AE3, p40, p63) and, in cases of suspected traumatic ulcerative granuloma with stromal eosinophilia (TUGSE), immunohistochemical stains to exclude lymphoproliferative disorders (e.g., CD3, CD20, CD30) as clinically indicated. Assessment was performed by an experienced head and neck pathologist (SA) within the institutional pathology department, following standardized diagnostic protocols. Histopathological diagnoses were categorized as benign or malignant based on pre‐specified routine histopathological diagnostic criteria and WHO classifications.

The pathologist evaluating the biopsy specimens had access to the relevant clinical information, as is standard practice, but was blinded to the ioUS findings. Indeterminate or non‐diagnostic ioUS results were excluded from quantitative analysis. For the reference standard, histopathological assessments with inconclusive diagnosis were excluded from the final analysis. All included lesions required definitive histopathological confirmation. Dysplastic lesions were excluded to ensure a clear binary between benign and malignant outcomes, thereby avoiding diagnostic ambiguity in this exploratory analysis. Cases with missing ioUS data or unavailable histopathological results were excluded from the final analysis. Benign lesions underwent excisional biopsy, while in cases of SCC, ioUS findings were correlated with the final surgical resection specimens obtained at a later stage following the initial biopsy.

As no established ioUS criteria exist for differentiating benign from malignant oral mucosal lesions, the definition of test positivity was determined *exploratory* within this study. Sonographic features including lesion size, margins, echogenicity, content, calcifications, and vascularity were assessed, and combinations of these features were analyzed to identify patterns associated with malignancy. The thresholds and feature combinations yielding the best diagnostic discrimination were used to define test positivity. Pre‐specified subgroup analyses were performed to explore potential sources of variability in diagnostic accuracy, including lesion size, borders, location, and histopathological subtype.

### 2.4 Statistical Analysis

Continuous variables were summarized using the median and compared using the Mann–Whitney test. Categorical variables were summarized with counts and percentages and compared using Fisher's exact test. Receiver operating characteristic (ROC) analyses was used exploratory to assess discriminatory ability of lesion's longest diameter as continuous variable. Logistic regression was used in an exploratory manner to examine associations between selected sonographic features and malignancy. Exploratory combinations of variables were examined descriptively based on the logistic regression odds ratio. Apparent discriminatory ability was assessed descriptively using the area under the curve (AUC). No internal or external validation was performed. A *p*‐value of less than 0.05 was considered statistically significant. Analyses were performed using R version 4.4.1 (R Foundation for Statistical Computing, Vienna, Austria).

## 3 Results

### 3.1 Clinical and Pathologic Characteristics

A total of 54 patients provided informed consent. Three patients were excluded: 1 patient died due to reasons unrelated to the study, 1 patient withdrew consent, and 1 patient did not undergo biopsy following ioUS. Thus, included were 51 patients (with 52 lesions), 24 females and 27 males, with a mean age 60.11 ± 16.7 years [range 18–90]. The median time between ioUS and biopsy was 3 days (range 0–14 days). Following histopathological results, 22 patients with 23 tumors were diagnosed with SCC, 3 patients with dysplasia (1 mild, 1 moderate, and 1 severe), 3 patients with non‐diagnostic pathology, and the remaining 23 patients with benign lesions (see Figure 2 for STARD flow diagram). Cases with non‐diagnostic pathology or dysplasia (which do not meet criteria for benign or malignant) were excluded, resulting in an analysis of 23 histologically confirmed SCC cases and 23 benign cases. The ioUS procedure was well tolerated by all participants, and no adverse events were recorded during its performance. One patient experienced transient lip numbness and persistent hypoesthesia following biopsy.

**FIGURE 2:**
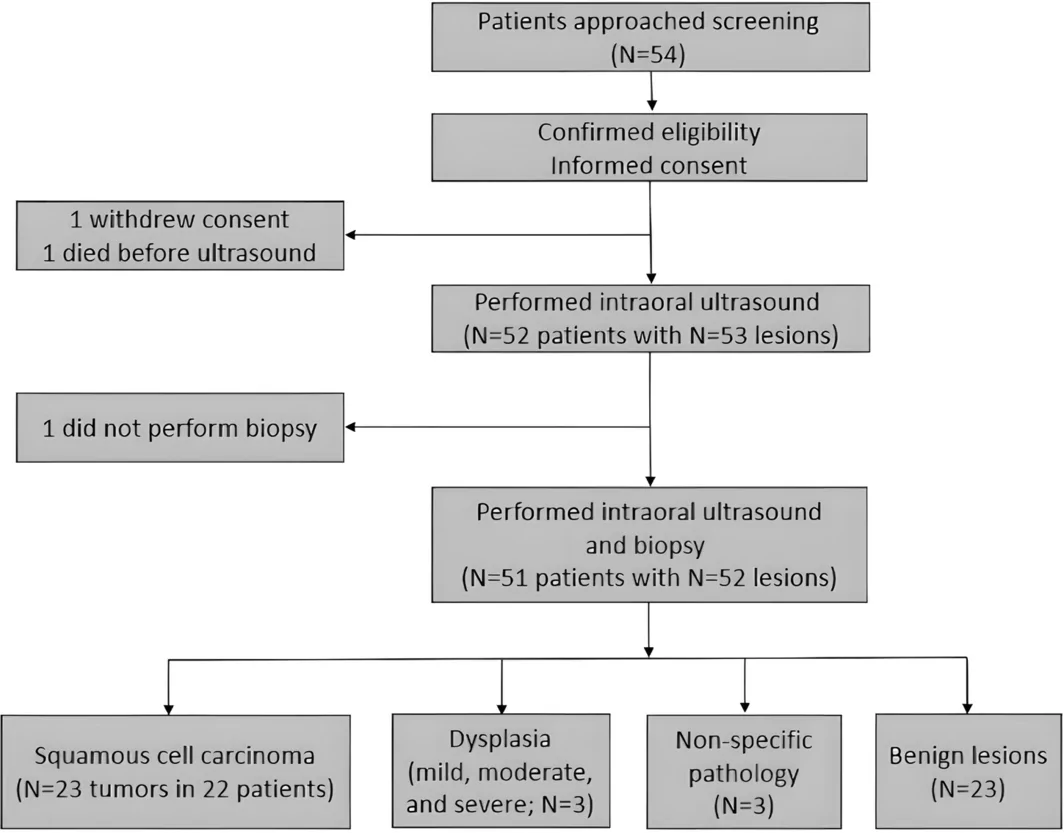
STARD flow diagram of patients in the study.

Most SCC tumors were located on the tongue (*n* = 15, one on dorsal tongue and 14 on the lateral tongue), followed by the floor of the mouth (*n* = 3) and lips (*n* = 3, 1 upper lip; 2 lower lip) and the buccal mucosa (*n* = 2, both in the cheek proper/main lining mucosa overlying the buccinator muscle). The majority were early‐stage, with 12 T1, 9 T2, and 2 T3 tumors. The clinical morphological appearances of SCC tumors were as follows: 19 cases presented as ulcers, 3 as white plaques, and 1 as red lesion. Benign lesions included fibroma (*n* = 5), minor salivary gland sialadenitis/mucoceles (*n* = 4), frictional hyperkeratosis (*n* = 4), pyogenic granuloma (*n* = 3), TUGSE (*n* = 2), and single cases of traumatic ulcer, epidermoid cyst, granular cell tumor, squamous papilloma, and lipoma. Minor salivary gland sialadenitis refers to inflammation of the minor salivary glands distributed throughout the oral mucosa, most commonly presenting as mucoceles (Kim et al. 2024). Less commonly, it may result from obstruction, autoimmune disease, or ischemic injury, with presentations ranging from painless swellings to ulcerative lesions that can mimic malignancy (Kim et al. 2024). TUGSE differs from traumatic ulcers by presenting as a slow‐healing, indurated ulcerative mass with rolled borders that can mimic malignancy (Chansaenroj et al. 2026), whereas traumatic ulcers resolve within 1–2 weeks after removing the cause. Histologically, TUGSE is characterized by dense eosinophilic infiltrate, deep tissue involvement, and atypical mononuclear cells, distinguishing it from traumatic ulcers (Chansaenroj et al. 2026).

The locations of benign lesions were as follows: tongue (*n* = 12; one on the dorsal tongue and 11 on the lateral tongue), lips (*n* = 5 all in the lower lip), floor of the mouth (*n* = 2), and buccal mucosa (*n* = 5, all in the cheek proper). The distribution of lesion locations is presented in Table 1. While there were differences in locations between the two groups, these differences did not reach statistical significance. The clinical morphological appearances of benign lesions included bumps or submucosal nodules (*n* = 12), white plaques (*n* = 4), ulcerated exophytic nodules (*n* = 3), ulcerated indurated masses (*n* = 2), a shallow ulcer (*n* = 1), and an exophytic white papillary growth (*n* = 1).

**TABLE 1:** Univariate analysis of clinical and sonographic variables comparing benign and SCC groups.

Parameter	Benign	Malignant (SCC)	*p*
**Location**			
Tongue	12	15	0.468
Lips/labial mucosa	5	3	
Floor of the mouth	2	3	
Buccal mucosa	5	2	
Gingiva	2	0	
**Sonographic variables**			
Maximal diameter (mm)	11.16 ± 8.41 [2.4–40]	16.42 ± 7.09 [2–32]	**0.005**
**Echogenicity**			
Hypoechoic	10 (58.8%)	18 (81.8%)	0.1582
Hyperechoic + mixed	7 (41.2%)	4 (18.2%)	
**Margin**			
Well‐defined	17 (100%)	3 (13.6%)	**< 0.001**
Ill‐defined	0 (0%)	19 (86.4%)	
**Content**			
Cystic	2 (12.5%)	0 (0%)	0.1707
Solid	14 (87.5%)	22 (100%)	
**Calcifications**			
Yes	5 (29.4%)	7 (31.8%)	1.00
No	12 (70.6%)	15 (68.2%)	
**Increased vascularity**			
Yes	4 (23.5%)	16 (76.2%)	**0.002**
No	13 (76.5%)	5 (23.8%)	
**Vascularity type**			
Type I	13 (76.5%)	5 (23.8%)	**0.006**
Type II	1 (5.9%)	3 (14.3%)	
Type III	3 (17.6%)	7 (33.3%)	
Type IV	0 (0%)	6 (28.6%)	
**Vascularity**			
Low (type I + II)	14 (82.4%)	8 (38.1%)	**0.008**
High (type III + IV)	3 (17.6%)	13 (61.9%)	

*Note:* Bold *p*‐values indicate statistical significance (*p* < 0.05).

Abbreviation: SCC, squamous cell carcinoma.

### 3.2 Features of Benign and SCC Groups

Figure 3 provides examples of clinical, ioUS, and histopathological images of a benign lesion (epidermoid cyst), an SCC case, and a reactive lesion (TUGSE). TUGSE typically appears as an ulcerated, indurated lesion that can mimic malignancy clinically, but it is a reactive self‐limiting condition (Misra et al. 2022).

**FIGURE 3:**
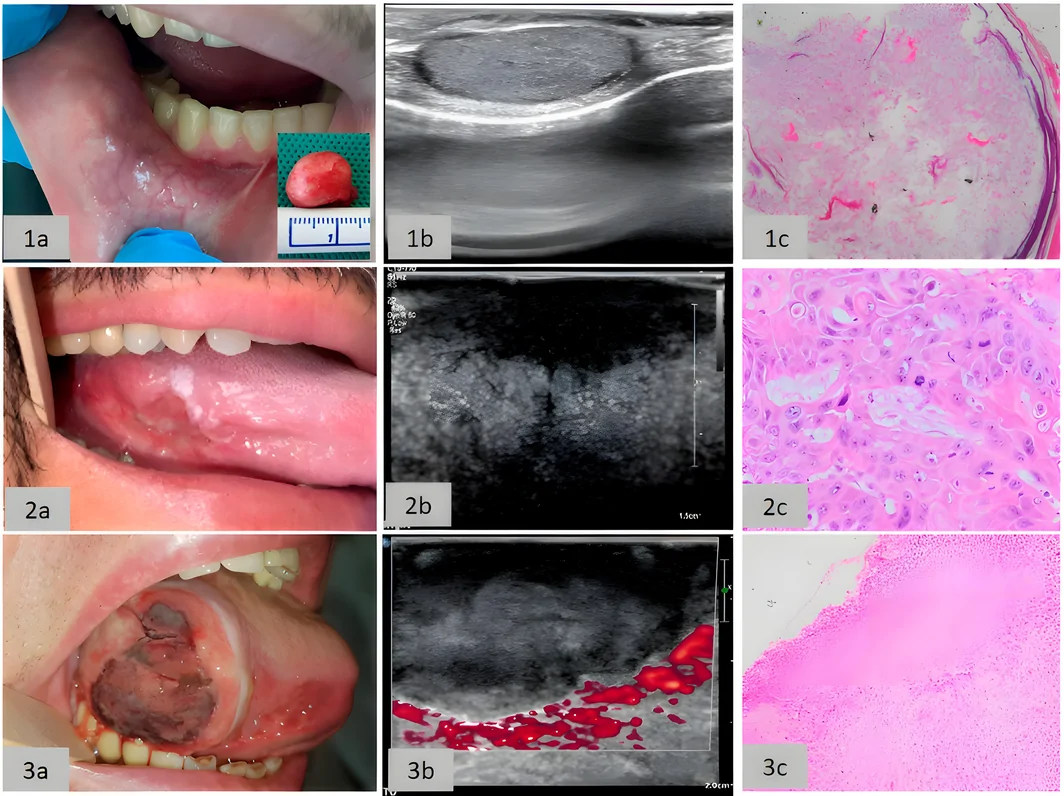
Examples of clinical images, intraoral ultrasound (ioUS) images and histopathology images of a benign lesion (1a–1c), a malignant tumor (2a–2c) and a reactive benign lesion, clinically suspected for malignancy (3a–3c). Case 1: A lump in the lower labial mucosa in an 18 year‐old male (1a), ioUS showed a well‐defined hyperechoic avascular lesion (1b). Histopathology showed an epidermoid cyst (1c), hematoxylin and eosin, ×40 magnification. Case 2: An ulcer of the lateral tongue in a 42 years‐old male (2a), ioUS showed an ill‐defined hypoechoic and hypervascular lesion (2b). On histopathology squamous cell carcinoma was evident (2c), hematoxylin and eosin, ×100 magnification. Case 3: An ulcerated mass of the lateral tongue clinically suspicious for malignancy in a 68 year‐old male (3a), ioUS showed a well‐defined, mixed echogenicity lesion, with peripheral vascularity (3b). Histopathology was consistent with traumatic ulcerative granuloma with stromal eosinophilia (TUGSE) (3c), hematoxylin and eosin, ×40 magnification. TUGSE is a reactive benign self‐limiting lesion, often misdiagnosed as malignancy based on clinical examination.

Sonographic variables including diameter, echogenicity, borders, content, calcifications, and vascularity were compared between benign and SCC groups by performing univariate analysis (Table 1). Among the sonographic variables, maximal diameter (*p* = 0.005), margin (*p* < 0.001), and vascularity (*p* = 0.002) proved to be significantly different between benign and SCC groups. Vascularity type (I, II, III, IV, and low vs. high) was also correlated with the SCC group (Table 1). Receiver operating characteristic (ROC) analysis was used to determine the best cutoff value for the longest diameter in differentiating between the two groups (Figure S1). As a result, the cutoff value was set at 12 mm. The sensitivity and specificity for this cutoff value were 0.76 (95% CI: 0.53–0.90) and 0.77 (95% CI: 0.57–0.90), respectively. The area under the curve (AUC) was 0.76 (95% CI: 0.60–0.92), indicating moderate discriminatory ability of this feature within this dataset (Figure S1).

Subsequently, exploratory multivariable analysis was performed to evaluate key sonographic parameters: maximal size, vascularity, and margins of each lesion (Table 2). However, margins could not be included in the multivariable analysis because all benign lesions had well‐defined margins, resulting in a null outcome and perfect separation. Similarly, type IV vascularity occurred only in malignant cases. Therefore, types III and IV were combined. In multivariate analysis, size > 12 mm and type III/IV vascularity were identified as associated with SCC (Table 2).

**TABLE 2:** Results of the exploratory multivariate analysis examining associations between sonographic features and outcome within this cohort.

Sonographic variable	Odds ratio	*p*	95% CI
**US maximal size**			
Less than 12 mm	Reference		
More than 12 mm	11.15	0.015	1.8–103.4
**Vascularity**			
Type I	Reference		
Type II	6.68	0.154	0.5–166.5
Type III/IV	8.48	0.050	1.13–94

Abbreviation: CI, confidence interval.

Based on these findings, combinations of sonographic features were explored descriptively to assess features of benign and malignant oral mucosal lesions (Table 3). For descriptive purposes, a composite feature pattern was examined wherein every case was given a score between 0 and 5 (Figure 4a). The mean ± standard deviation of the benign and SCC group scores were 0.6 ± 0.9 and 4.1 ± 1.5, respectively. None of the benign cases received a score of four or five, as the figure illustrates. Using four as a threshold value, the sensitivity was 0.85 (95% CI: 0.65–0.95), the specificity was 1.00 (95% CI: 0.81–1.00), and the AUC was 0.92 (95% CI: 0.84–1.00) (Figure 4b). However, given that feature selection and evaluation were performed on the same small cohort without validation, these estimates are likely optimistically biased and should be interpreted as exploratory. The limited sample size further increases the risk of overfitting, particularly for analyses involving combinations of variables, and all findings should be interpreted with caution.

**TABLE 3:** Exploratory combinations of variables in this cohort, combined based on their respective odds ratios from the logistic regression analysis.

Sonographic risk factor	Score
**US maximal size**	
Less than 12 mm	0
More than 12 mm	2
**Vascularity**	
Type I/II	0
Type III/IV	1
**Borders**	
Well‐defined	0
Ill‐defined	2

*Note:* This approach allows for a descriptive stratification of cases according to the density of how sonographic features were distributed across the lesions:
Lower Association (0–1): Minimal presence of sonographic features of interest. Intermediate Association (2–3): Moderate presence of sonographic features. Stronger Association (4–5): High concentration of sonographic features correlated with malignancy in this cohort.
This categorization is purely exploratory and serves to illustrate feature distribution patterns within the study sample rather than providing a validated predictive model.

**FIGURE 4:**
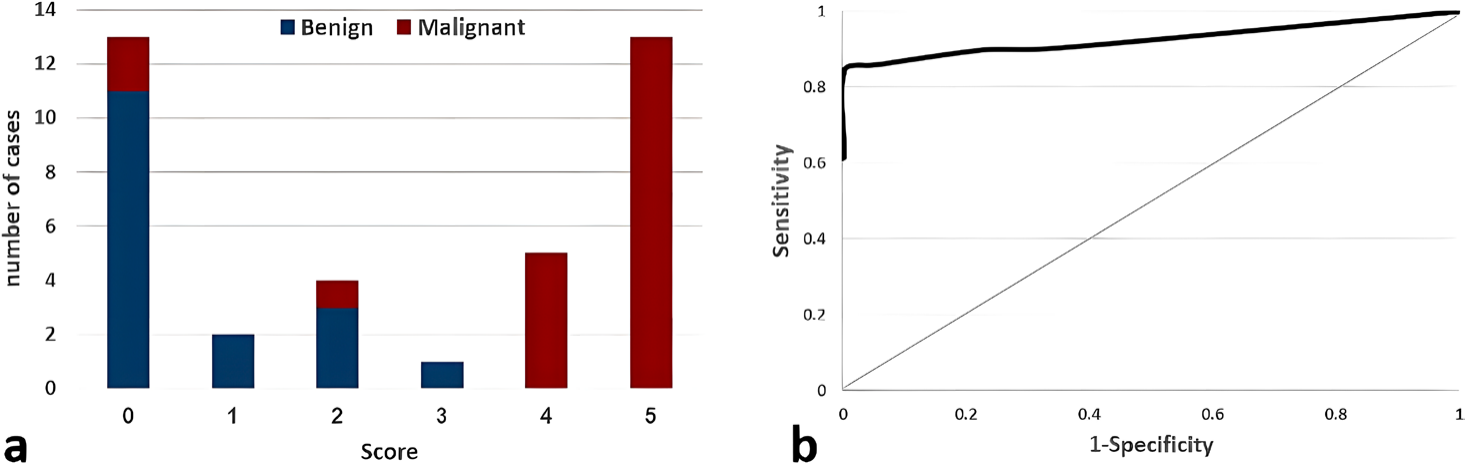
Exploratory representation of combined sonographic features in benign and squamous cell carcinoma (SCC) lesions. (a) Distribution of composite feature scores across cases. The number of cases and corresponding composite scores are displayed on the *X* and *Y* axes, respectively. No benign cases were observed with scores of 4 or 5 in this cohort. (b) Receiver operating characteristic (ROC) curve illustrating the apparent discriminatory ability of the composite feature pattern within the same dataset. A threshold of 4 is shown for descriptive purposes. The area under the curve (AUC = 0.92; 95% CI: 0.84–1.00) reflects performance within this cohort and should be interpreted cautiously, as feature selection and evaluation were performed on the same dataset without validation.

## 4 Discussion

This pilot study demonstrates ioUS features across benign and malignant oral lesions and explores combinations of sonographic features associated with malignancy that warrant validation in larger, more diverse cohorts. Accurate differential assessment is important for appropriate clinical management. In this context, ioUS may offer a supplementary means of localizing and characterizing areas of potential clinical concern. US also provides several practical advantages, including real‐time imaging, bedside use, and broad availability, while avoiding ionizing radiation and allowing safe repeated examinations.

Although US is widely used for evaluating soft tissue lesions in other regions of the body, its application in the oral cavity remains limited. Only a small number of studies have reported on the use of ioUS for the assessment of oral soft tissue lesions (Derindağ et al. 2021; Joshi et al. 2014; Konishi et al. 2023; Pacheco‐Pereira et al. 2025). While these studies provide important preliminary insights, prospective evaluations of sonographic features for the pre‐operative differentiation between benign and malignant oral cavity tumors are still relatively sparse. Further investigation in this area may help refine diagnostic accuracy and support treatment planning in oral oncology.

Previous publications indicate various benefits of US in the identification of soft tissue lesions outside the oral cavity (Takeshi Morii et al. 2018; Shu et al. 2022). US has several advantages: it is fast, accurate, available, well tolerated by patients, cost‐effective, and safe (no ionizing radiation). Furthermore, US has superiority in imaging small tumors in the oral cavity that other diagnostic imaging modalities like CT and MRI may not detect, unrelated to the presence of metal artifacts (Marcello Scotti et al. 2022). Based on whether the tumor was visible or not during imaging, previous studies made the observation that oral tumors of less than 4–5 mm are not detectable on CT or MRI (Baba et al. 2019; Shintani et al. 2001), whereas US can detect tumor thickness as small as 1–2 mm, as demonstrated also in the present study. Nevertheless, the presence of dental metallic materials may introduce imaging artifacts not only in MRI and CT but also in US, including posterior acoustic shadowing, comet‐tail artifacts, and reverberation artifacts (Yuce and Acikgoz 2025). Recognition of these artifact patterns is important to ensure accurate image interpretation and to avoid misattribution of artifact‐related findings to true pathology.

Sonographic features including maximal diameter (T. Morii et al. 2018; Nagano et al. 2015; Oebisu et al. 2014), hypoechogenicity (Belli et al. 2000; Nagano et al. 2015), ill‐defined borders (Belli et al. 2000; T. Morii et al. 2018) and increased vascularity (Belli et al. 2000; Giovagnorio et al. 1999; T. Morii et al. 2018; Oebisu et al. 2014) were previously reported to reliably differentiate between benign and malignant soft tissue tumors outside the oral cavity. However, no clear consensus exists regarding which US criteria are most reliable for this purpose. In the present study, three parameters, maximal diameter, lesion margins, and vascularity, emerged as sonographic features associated with malignancy in oral cavity lesions, consistent with findings reported for soft tissue tumors in other anatomical sites. All cases of malignant lesions in this study were SCC. Sonographic features of skin SCC are generally described as ill‐defined, hypoechoic, and vascular (Valian et al. 2024; Wortsman 2024), mirroring findings in oral SCC cases in this cohort. Using ROC analysis to develop a theoretically derived threshold, the 12 mm cutoff for maximal diameter provided the highest sensitivity and specificity. In other reported soft tissues, this cutoff was usually around 5 cm (T. Morii et al. 2018; Nagano et al. 2015). In the present study, no consistent differences in echogenicity, calcifications, or cystic components were observed between benign lesions and SCC.

Interestingly, ill‐defined margins in soft tissue tumors beyond the oral region are reported to be relatively uncommon. For instance, Morii et al. (2018) observed that the prevalence of ill‐defined margins in benign and malignant soft tissue tumors was 7.1% and 25%, respectively. Other studies also showed a similar pattern, with ill‐defined margins in benign tumors to be 10% (Oebisu et al. 2014) and 22% (Belli et al. 2000), and in malignant tumors to be 32.2% (Oebisu et al. 2014) and 45% (Belli et al. 2000). Remarkably, in the current study, ill‐defined margins were not encountered in any case of benign tumors, but were observed in 86.4% of malignant SCC cases.

Tumor vascularity seems to be a consistent factor in many reports for differentiating benign from malignant soft tissue tumors, usually utilizing the approach developed by Giovagnorio (Giovagnorio et al. 1999) that was based on data from skin cancer. Because pre‐angiogenic factors are up‐regulated in cancerous tumors, there is enhanced angiogenesis (Viallard and Larrivée 2017). Indeed, vascularity is one of the most often cited characteristics in previous research when assessing the malignancy of soft tissue masses (Belli et al. 2000; Giovagnorio et al. 1999; T. Morii et al. 2018; Oebisu et al. 2014). Furthermore, some studies assigned vascularity a higher value than other characteristics when developing scoring systems for differentiating between benign and malignant soft tissue masses (T. Morii et al. 2018; Nagano et al. 2015).

In studies employing UHFUS for the evaluation of oral lesions, mucosal thickness and vascularity have been identified as key sonographic parameters for lesion characterization (Izzetti, Vitali, Aringhieri, Oranges, et al. 2020). Mucosal thinning reflects epithelial atrophy characteristic of SCC and in several inflammatory and autoimmune conditions. Likewise, pronounced vascularity has been associated with malignant lesions (Izzetti, Vitali, Aringhieri, Caramella, et al. 2020a; Izzetti, Vitali, Aringhieri, Oranges, et al. 2020), in line with the present findings. These associations are biologically plausible, as they mirror underlying dysplastic and angiogenic processes (Fogante et al. 2022; Izzetti, Vitali, Aringhieri, Oranges, et al. 2020).

Combinations of key sonographic features were explored descriptively from the identified sonographic features associated with malignant oral mucosal lesions in this cohort. A simple composite representation was examined for illustrative purposes where each case was assigned a score ranging from 0 to 5, calculated by summing the individual scores of the relevant ioUS variables. Low scores (0–1) were more observed in benign lesions, whereas high scores (4–5) were more observed in SCC lesions. Although a low score may be suggestive of a benign lesion, this composite approach was not developed or validated as a predictive model and should not be used for clinical decision‐making. Therefore, all suspicious lesions should be biopsied in accordance with professional standard practice.

While experienced clinicians can often diagnose common benign oral lesions without imaging, the aim of this study was not to replace clinical judgment or propose ioUS as a standalone diagnostic alternative. Rather, the study was methodological and exploratory in nature, focusing on the identification of sonographic features that may inform the development of future adjunctive risk‐stratification tools.

The benign comparator group was included as a biological contrast to facilitate feature selection and the construction of an exploratory framework. While this approach may limit immediate clinical applicability, it provides a necessary foundation for future studies aimed at refining sonographic criteria, validating thresholds, and integrating ioUS findings with clinical assessment. Accordingly, the proposed framework is presented as hypothesis‐generating and strictly complementary, intended to support, not replace, clinical decision‐making and histopathological confirmation.

Moreover, several sonographic features, including size and vascularity, are not specific to malignancy and may also be present in benign conditions. In this study cohort, for example, pyogenic granulomas and TUGSE lesions demonstrated marked vascularity, whereas small or early SCC tumors showed low vascularity: two cases of SCC assigned a score of 0 (Figure 4a). The first case involved a lip SCC presenting as an ulcer (7 × 4 × 2 mm, DOI = 2 mm), with well‐defined borders on ioUS and no detectable vascularity. The second was a lateral tongue SCC presenting as a white plaque (8 × 8 × 1.5 mm, DOI = 1.5 mm), also with well‐defined borders and absent vascularity. While these two cases are not representative of broader cohort trends, they serve to demonstrate that neither individual nor combined sonographic features are specific to one diagnostic category.

This overlap underscores the need for cautious interpretation of ioUS findings within the broader clinical context and reinforces that features such as increased vascularity and size should be regarded as context‐dependent indicators rather than definitive markers of malignancy. Accordingly, the findings should be viewed as exploratory tools for pattern recognition and hypothesis generation, not as an independent diagnostic approach.

While encouraging, this study has several limitations. First, the small sample size has limited statistical power, potentially reducing the ability to detect subtle differences in sonographic features and restricting the generalizability of the results across diverse patient populations and lesion characteristics. The small sample size and the use of multivariable analysis in this context introduce a high risk of overfitting. The absence of internal or external validation further limits confidence in the stability and generalizability of the observed associations. Additionally, thresholds and feature combinations were derived and evaluated within the same dataset, which likely results in optimistic bias in reported performance metrics. Accordingly, larger prospective multicenter studies are warranted.

Secondly, there may be selection bias because all included malignant cases were SCC. If other malignant entities of the oral cavity were included, the significant sonographic parameters might change. Since SCC constitutes around 90%–95% of malignant tumors in the oral cavity, this drawback might be mitigated in a much larger series.

Thirdly, it is important to keep in mind that no single or combined sonographic features can precisely differentiate between benign and SCC tumors. In fact, in the present study there were some SCC tumors as small as 1.5 mm with low vascularity, and reactive benign lesions such as TUGSE as large as 4 cm with high vascularity. This overlap underscores the importance of interpreting ioUS findings in the context of clinical and histopathological information, rather than relying solely on isolated imaging features. To overcome this, future research should assess larger cohorts and additional sonographic features for tissue characterization in the oral cavity, such as vascular morphology, vessel density and organization, flow velocity (Griffith et al. 2004), and contrast enhancement with US contrast agents (Du et al. 2012). Elastography was not assessed in this pilot study due to equipment limitations and the exploratory nature of the work, and is therefore acknowledged as a limitation and identified as a priority for future research.

Importantly, the proposed role of ioUS is not to replace clinical assessment or histopathological confirmation but to provide complementary information in the pre‐biopsy setting. As a non‐invasive modality, ioUS may contribute to more targeted biopsy approaches in selected patients, with future large‐scale validation required to define its role.

Within current diagnostic pathways, the identification of specific ioUS features is intended to serve as a supplementary clinical aid, with these descriptive associations intended to offer additional insight into the nature of oral mucosal lesions. This approach may be particularly valuable in patients with equivocal clinical findings, extensive mucosal involvement, those requiring multiple or repeated biopsies, or when submucosal characteristics cannot be reliably assessed by clinical inspection or palpation alone, where optimized targeting could improve diagnostic yield and reduce procedural burden. With further validation in larger, stratified cohorts, such a framework could be incorporated into routine assessment algorithms to complement clinical examination and histopathological evaluation.

While this descriptive ioUS approach appears feasible for point‐of‐care assessment, it should be regarded as exploratory and hypothesis‐generating and further investigation involving larger, more diverse datasets is required to fully characterize the sonographic variety of oral lesions. Additionally, perfect separation for some variables (e.g., lesion margins) limited their inclusion in the multivariate analysis and may have influenced the derived descriptive scores. Future studies should consider approaches such as penalized regression or bootstrapping to mitigate potential bias. The study did not include internal validation (e.g., cross‐validation or resampling), which limits confidence in the stability and generalizability of the reported AUC and cutoff values, and these findings should therefore be interpreted with caution. Merging vascularity types III and IV, while methodologically necessary, may mask subtle differences in vascular patterns. With more extensive datasets, it may become possible to analyze type III and IV vascularity separately, which could highlight patterns not detectable in the current study. Histopathological assessment in some benign cases (e.g., TUGSE) was based on incisional biopsy rather than whole‐lesion evaluation. This may limit the accurate assessment of parameters such as dimensions including depth, margin status, and intralesional heterogeneity. Ongoing research aims to further investigate these descriptive sonographic features and refine the overall approach to their assessment. Future directions may include machine learning algorithms trained on large datasets of ioUS images, which could automate lesion classification and support clinical decision‐making in real‐time, even for less experienced operators.

Most SCC cases in this cohort presented clinically as ulcers, a feature that may have influenced sonographic characteristics, particularly vascularity. The cohort did not include clinically relevant SCC morphologies beyond ulcerative presentations, such as nodular, exophytic, and mixed lesions. Clinical lesion morphology of benign lesions was not systematically evaluated and may represent an additional confounding factor affecting sonographic findings. Furthermore, the limited representation of high‐risk potentially malignant disorders restricts applicability to borderline diagnostic scenarios, which often present the greatest clinical challenge. While frictional keratosis cases met inclusion criteria, we acknowledge the potential for histopathological overlap with low‐grade dysplasia, which may introduce selection bias. The scope of these exploratory findings is further restricted by the lack of representation from anatomical sites such as the palate, gingiva, and ventral tongue. In addition, the study did not compare sonographic features between lesions of similar morphology or anatomical site. Secondary infection and topical inflammatory changes were not systematically assessed and may have influenced sonographic appearances in both benign and malignant lesions. Accordingly, the descriptive sonographic features should be considered developmental, and validation in larger and more diverse cohorts enriched with high‐risk potentially malignant disorders, stratified by SCC specific grades is necessary before broader clinical application.

Finally, limitations of ioUS include that it is operator dependent and requires experience, so the presented results may not be replicated with other operators. All ioUS examinations were performed by a single highly experienced radiologist using a standardized acquisition protocol and predefined criteria to minimize subjectivity and ensure internal consistency; nevertheless, inter‐ and intra‐operator variability were not assessed, limiting generalizability. Future studies should assess inter‐ and intra‐operator variability to establish the reproducibility of ioUS findings in broader clinical practice. It should be noted that the radiologist had extensive experience in general US but no prior experience with ioUS. Furthermore, although efforts were made to blind the US operator to clinical information, complete blinding in a real‐world clinical setting may not be fully achievable, and this should be considered a potential source of bias when interpreting the findings. The type of coupling medium can affect sonographic appearance and tissue measurements, as saliva alone is generally inadequate; commercial oral gels are recommended to minimize soft‐tissue compression and optimize image quality. Another limitation is that areas such as anterior floor of mouth are difficult or impossible to scan with the conventional hockey stick probe, so access to some areas in the oral cavity is restricted. Innovative probe designs are a potential solution to this problem and are being explored.

## 5 Conclusions

This prospective exploratory pilot study evaluated sonographic features in a small cohort of benign and malignant oral lesions using ioUS. Specific characteristics, such as a tumor diameter exceeding 12 mm, ill‐defined margins, and increased vascularity, were more frequently observed in malignant cases. While these preliminary findings suggest potential associations between certain sonographic patterns and malignancy, they are intended to serve as a foundation for future hypothesis‐driven research. Given the pilot nature of this work, any clinical application is premature and necessitates further evaluation in larger, more diverse cohorts.

## Author Contributions

**Ragda Abdalla‐Aslan:** conceptualization, investigation, writing – original draft, methodology, visualization, writing – review and editing, project administration, supervision, data curation, resources. **Diana Gaitini:** conceptualization, visualization, writing – review and editing, project administration. **Adi Rachmiel:** investigation, writing – review and editing, project administration. **Goni Merhav:** investigation, writing – review and editing. **Sharon Akrish:** investigation, writing – review and editing. **Marcia Javitt:** conceptualization, writing – review and editing. **Dekel Shilo:** investigation, writing – review and editing. **Omri Emodi:** investigation, writing – review and editing. **Yotam Shkedy:** investigation, writing – review and editing. **Nira Beck‐Razi:** conceptualization, investigation, writing – review and editing, methodology, project administration, resources.

## Funding

The authors have nothing to report.

## Ethics Statement

All procedures were performed in compliance with the Helsinki declaration and institutional guidelines and have been approved by the institutional review board (RMB‐19‐0596).

## Consent

Each participant in this study signed informed consent to participate.

## Conflicts of Interest

The authors declare no conflicts of interest.

## Supporting information


**Figure S1:** Receiver operating characteristic (ROC) curve to determine the cutoff value of the longest diameter in discriminating between benign and SCC tumors. The most useful cutoff value was determined to be 12 mm. The area under the curve (AUC) was 0.76, suggesting that this cutoff value was useful for determining the malignancy of the tumors.

## Data Availability

The full study protocol, datasets used and/or analyzed during the current study are available from the corresponding author on reasonable request.
