# Supplementary material for: Pre‐Operative Intraoral Ultrasound Features of Benign and Malignant Oral Lesions: An Exploratory Pilot Study
Source: Oral Dis. 2026 Jun 18;32(10):3138–49. doi: 10.1111/odi.70395 (PMC13768907; doi:10.1111/odi.70395)
Supplement: Supplementary file 1 [file ODI-32-3138-s001.docx]

**Supplementary figure 1:** Receiver operating characteristic (ROC) curve to determine the cutoff value of the longest diameter in discriminating between benign and SCC tumors. The most useful cutoff value was determined to be 12 mm. The area under the curve (AUC) was 0.76, suggesting that this cutoff value was useful for determining the malignancy of the tumors.
